# Supplementary figures and images for: Soymilk residue (okara) as a natural immobilization carrier for Lactobacillus plantarum cells enhances soymilk fermentation, glucosidic isoflavone bioconversion, and cell survival under simulated gastric and intestinal conditions
Source: PeerJ. 2016 Nov 10;4:e2701. doi: 10.7717/peerj.2701 (PMC5111894; doi:10.7717/peerj.2701)

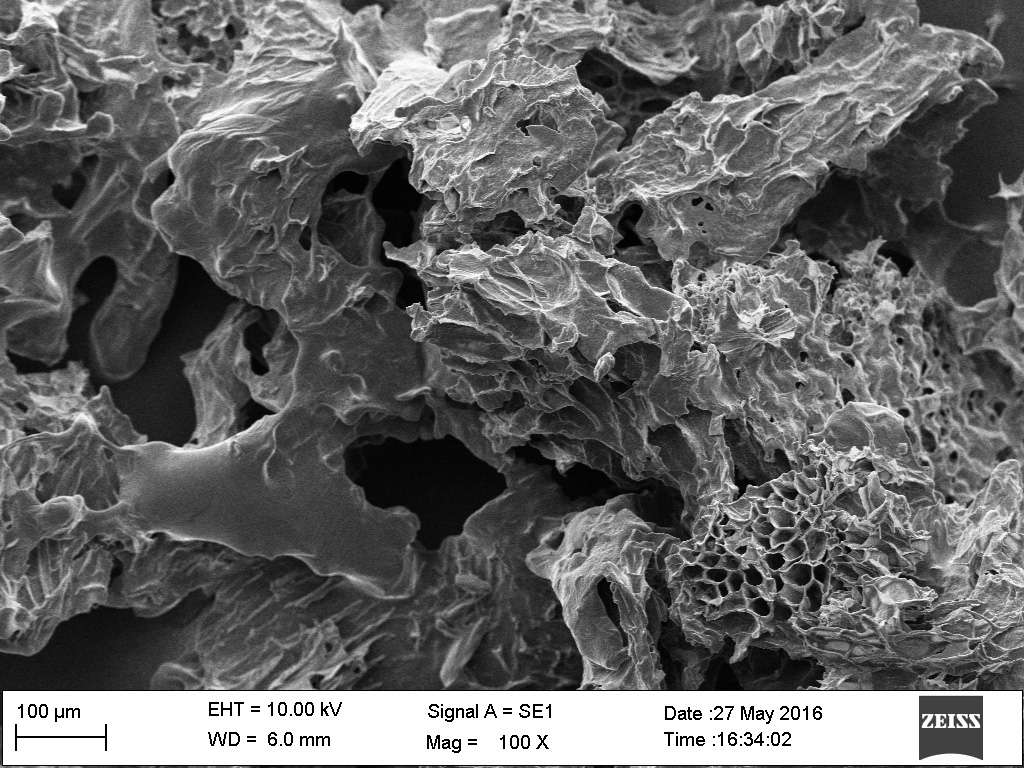

Supplement: Data S1 [file peerj-04-2701-s001.jpg]

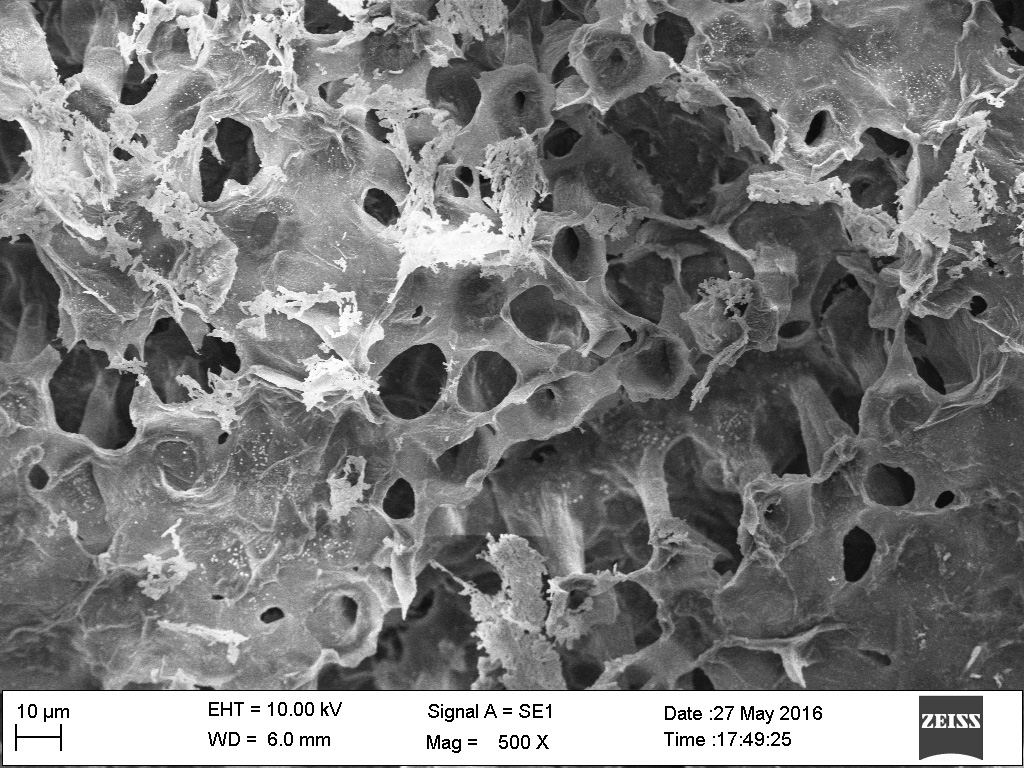

Supplement: Data S2 [file peerj-04-2701-s002.jpg]

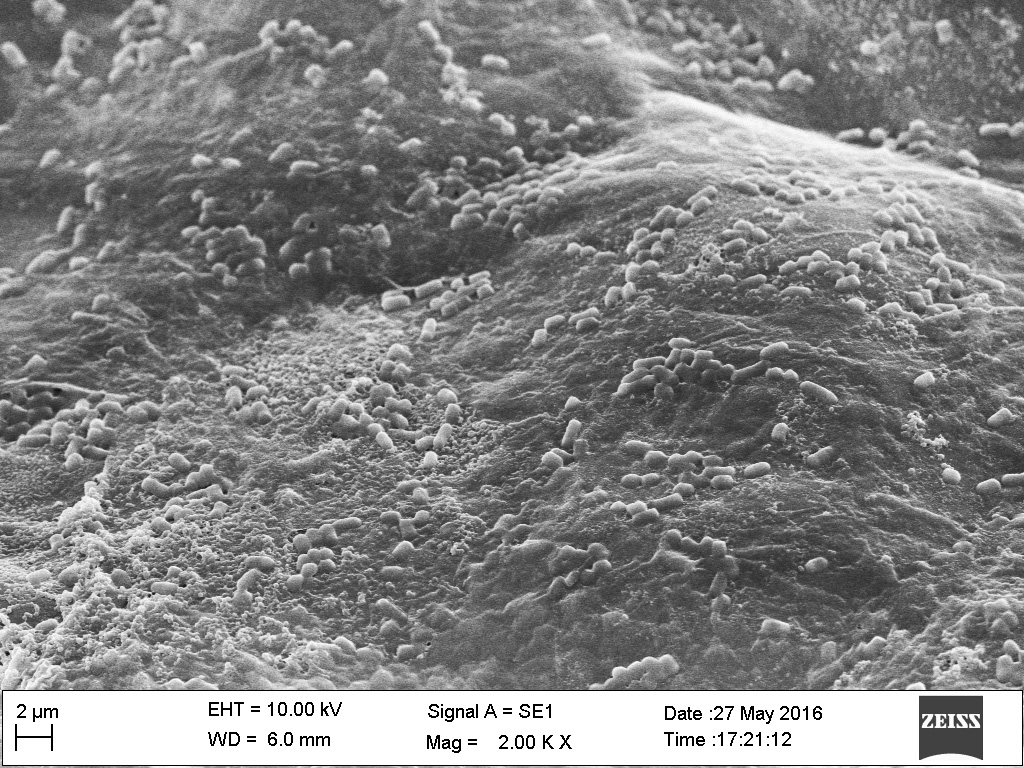

Supplement: Data S3 [file peerj-04-2701-s003.jpg]

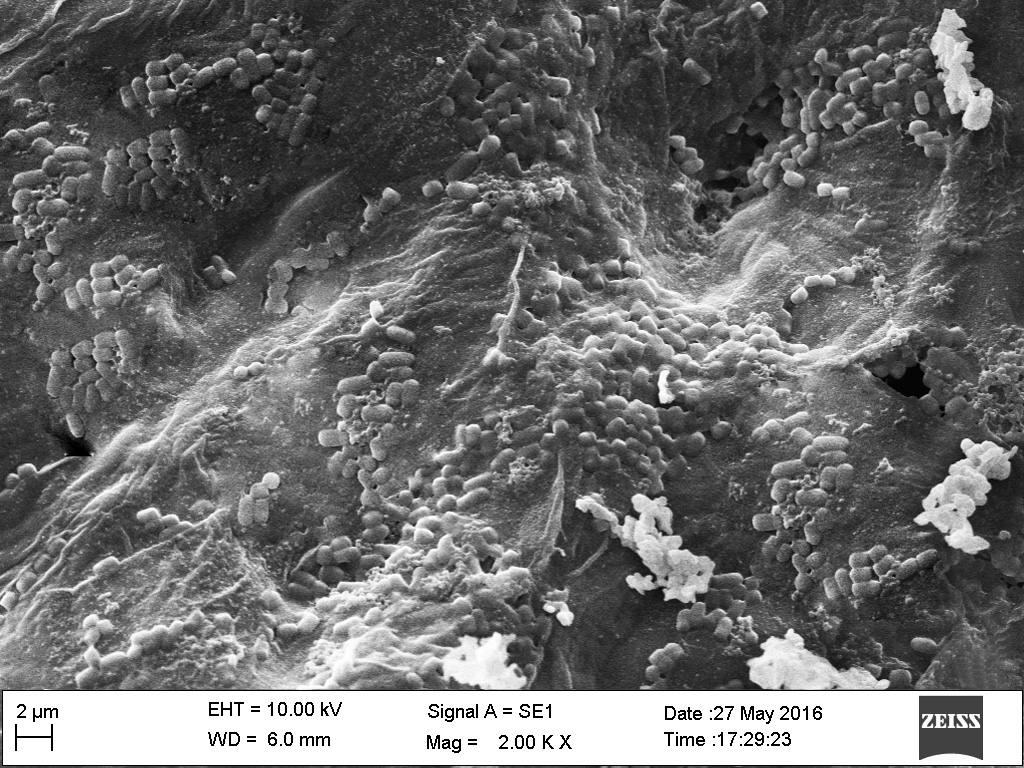

Supplement: Data S4 [file peerj-04-2701-s004.jpg]

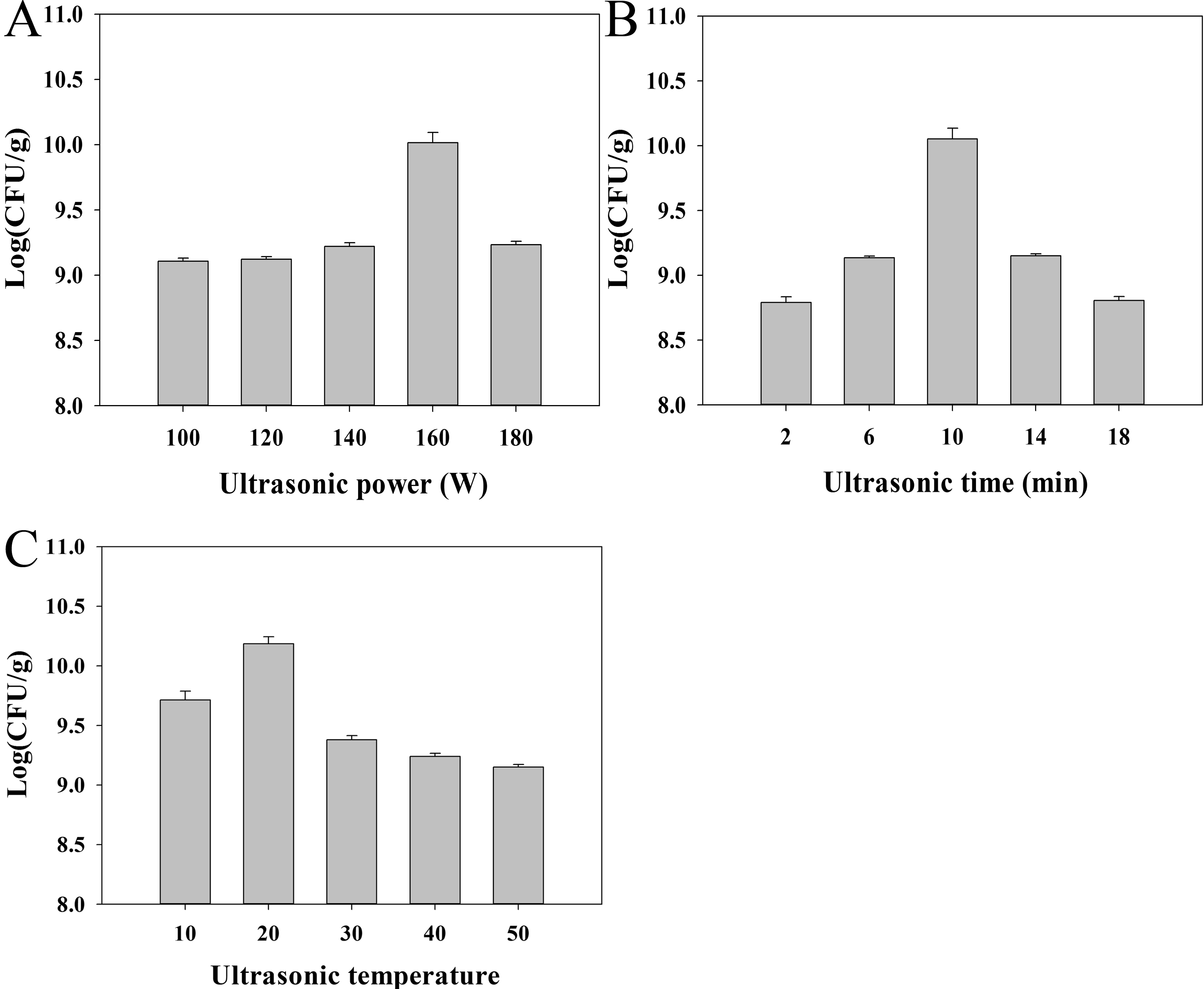

Supplement: Figure S1 — A, cells shedding from okara under different ultrasound power for 6 min at initial temperature of 10 °C. B, cells shedding from okara under ultrasound power of 160W for different time at initial temperature of 10 °C. C, cells shedding from okara under ultrasound power of 160W for 10 min at different initial temperature. CFU: colony forming units. [file peerj-04-2701-s012.png]
