# Supplementary material for: Soymilk residue (okara) as a natural immobilization carrier for Lactobacillus plantarum cells enhances soymilk fermentation, glucosidic isoflavone bioconversion, and cell survival under simulated gastric and intestinal conditions
Source: PeerJ. 2016 Nov 10;4:e2701. doi: 10.7717/peerj.2701 (PMC5111894; doi:10.7717/peerj.2701)
Supplement: Data S8 — Liquid chromatogram and raw data for analysis of isoflavone content of soymilk inoculated with free and immobilized L. plantarum 70810. FL: free L. plantarum 70810; IL:okara-immobilized L. plantarum 70810. [file peerj-04-2701-s008.docx]

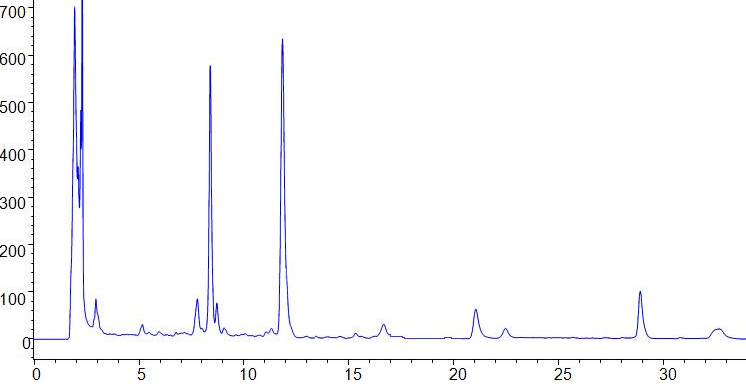


2

4

3

1


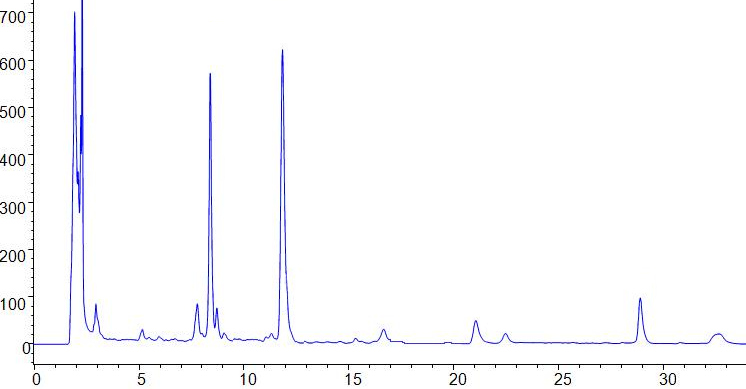


4

3

2

1


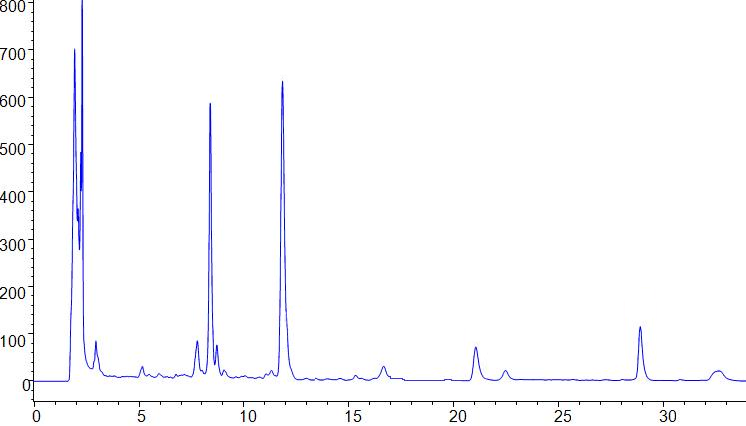


1

2

3

4

High-performance liquid chromatogram (HPLC) of isomeric isoflavones in extracts from unfermented soymilk. (1 = daidzin; 2 = genistin; 3 =daidzein; 4 = genistein).


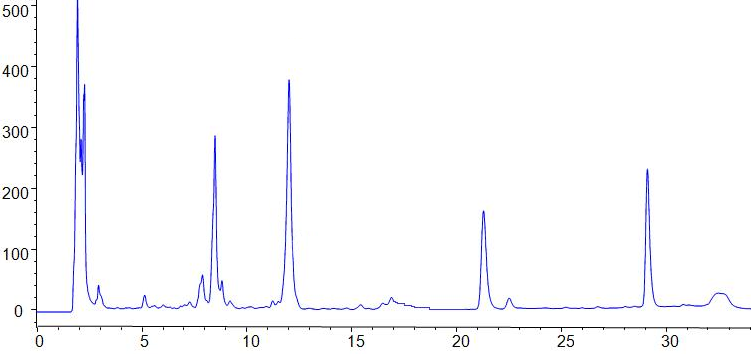


4

3

2

1


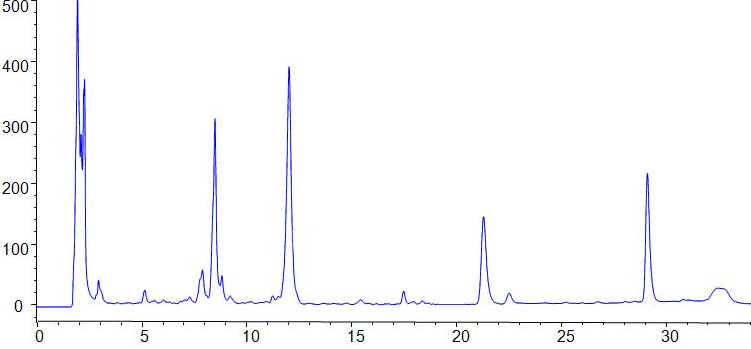


1

2

3

4


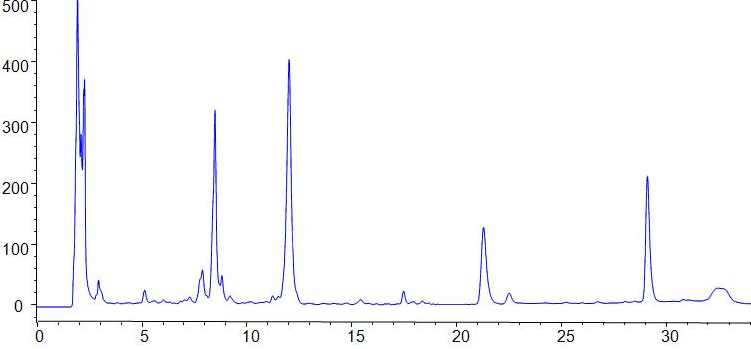


High-performance liquid chromatogram (HPLC) of isomeric isoflavones in extracts from soymilk fermented by free *L. plantarum 70810* for 4 hours. (1 = daidzin; 2 = genistin; 3 =daidzein; 4 = genistein).


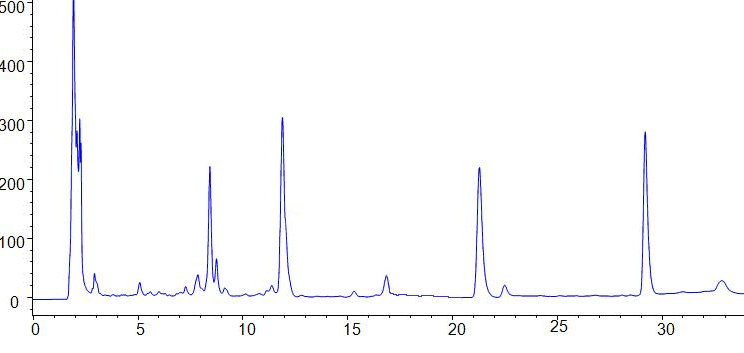

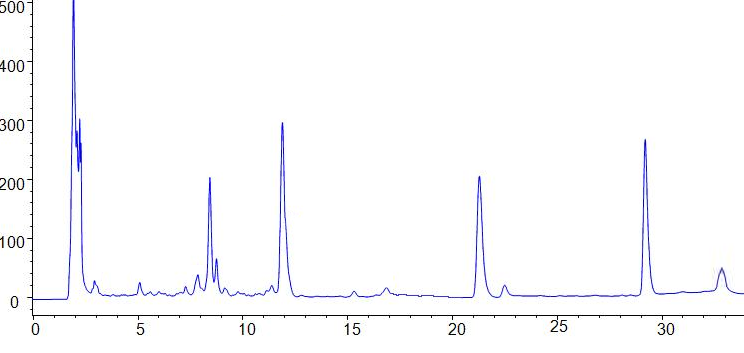


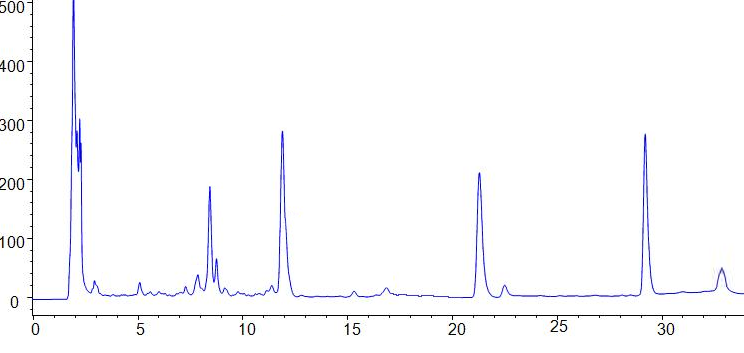


High-performance liquid chromatogram (HPLC) of isomeric isoflavones in extracts from soymilk fermented by free okara-immobilized *L. plantarum 70810* for 4 hours. (1 = daidzin; 2 = genistin; 3 =daidzein; 4 = genistein).


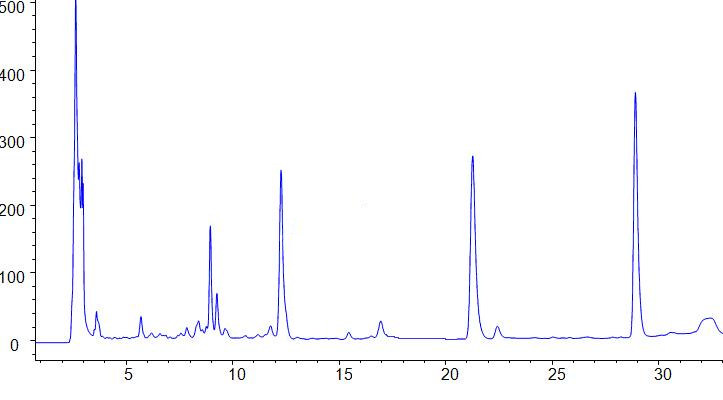


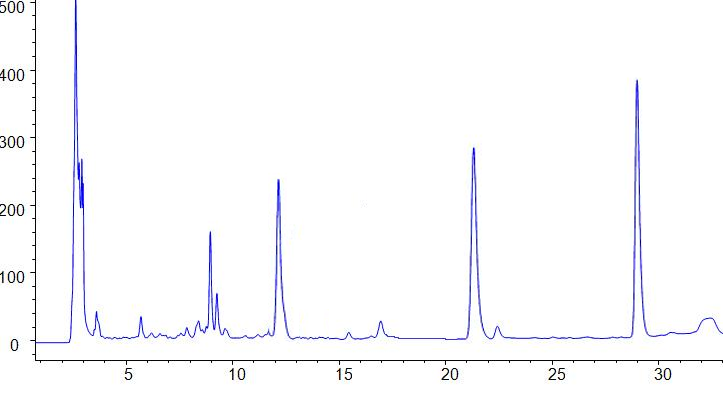

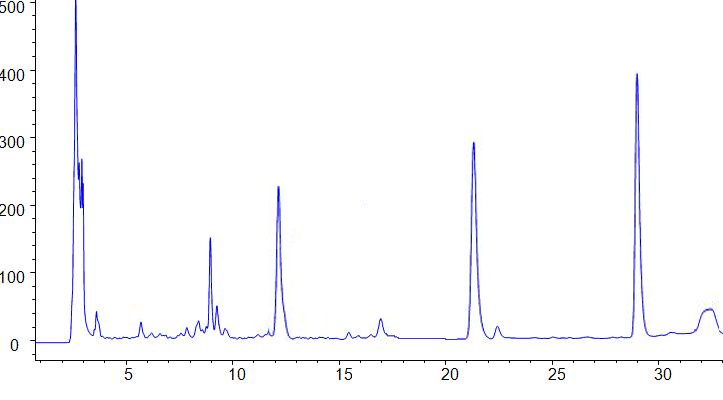


High-performance liquid chromatogram (HPLC) of isomeric isoflavones in extracts from soymilk fermented by free *L. plantarum 70810* for 8 hours. (1 = daidzin; 2 = genistin; 3 =daidzein; 4 = genistein).


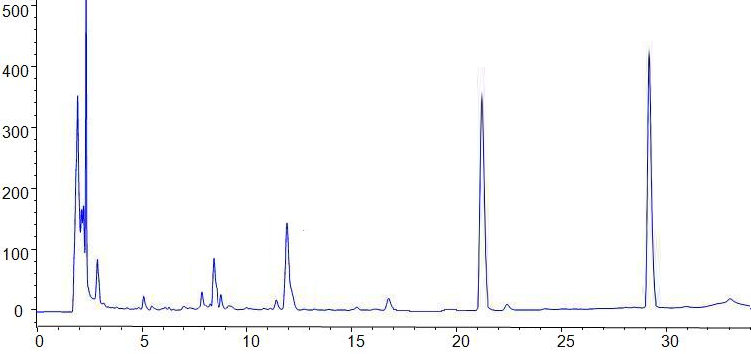


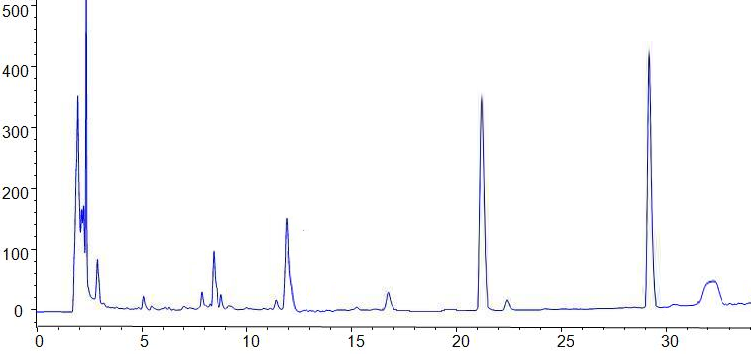

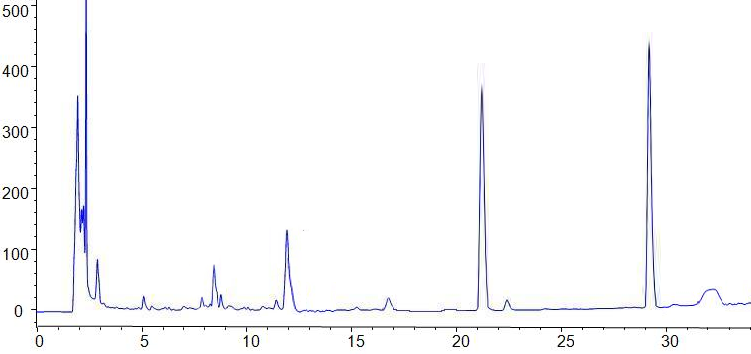


High-performance liquid chromatogram (HPLC) of isomeric isoflavones in extracts from soymilk fermented by free okara-immobilized *L. plantarum 70810* for 8 hours. (1 = daidzin; 2 = genistin; 3 =daidzein; 4 = genistein).

| Daidzin（μg/mL） | |  |  |  |  |  |  |  |  |
| --- | --- | --- | --- | --- | --- | --- | --- | --- | --- |
| FL |  |  |  |  |  | IL |  |  |  |
| Time(h) |  |  |  |  |  | Time(h) |  |  |  |
| 0 | 43.661 | 40.894 | 49.128 |  |  | 0 | 44.315 | 40.99 | 50.64 |
| 4 | 27.889 | 30.575 | 32.261 |  |  | 4 | 20.877 | 18.893 | 22.861 |
| 8 | 13.837 | 11.995 | 13.679 |  |  | 8 | 5.853 | 5.276 | 6.43 |
|  |  |  |  |  |  |  |  |  |  |
|  |  |  |  |  |  |  |  |  |  |
| Genistin（μg/mL） | |  |  |  |  |  |  |  |  |
| FL |  |  |  |  |  | IL |  |  |  |
| Time(h) |  |  |  |  |  | Time(h) |  |  |  |
| 0 | 68.631 | 60.457 | 67.805 |  |  | 0 | 66.155 | 63.061 | 72.249 |
| 4 | 44.556 | 46.395 | 47.717 |  |  | 4 | 31.95 | 26.918 | 29.934 |
| 8 | 21.936 | 19.147 | 19.725 |  |  | 8 | 6.51 | 6.779 | 7.441 |
|  |  |  |  |  |  |  |  |  |  |
|  |  |  |  |  |  |  |  |  |  |
| Daidzein（μg/mL） | |  |  |  |  |  |  |  |  |
| FL |  |  |  |  |  | IL |  |  |  |
| Time(h) |  |  |  |  |  | Time(h) |  |  |  |
| 0 | 3.1721 | 2.9571 | 3.2871 |  |  | 0 | 3.383 | 2.811 | 3.055 |
| 4 | 10.812 | 10.959 | 13.665 |  |  | 4 | 15.254 | 16.906 | 18.602 |
| 8 | 24.624 | 22.863 | 26.385 |  |  | 8 | 33.192 | 28.98 | 31.404 |
|  |  |  |  |  |  |  |  |  |  |
|  |  |  |  |  |  |  |  |  |  |
| Genistein（μg/mL） | |  |  |  |  |  |  |  |  |
| FL |  |  |  |  |  | IL |  |  |  |
| Time(h) |  |  |  |  |  | Time(h) |  |  |  |
| 0 | 4.797 | 3.913 | 4.481 |  |  | 0 | 4.337 | 4.119 | 4.655 |
| 4 | 15.873 | 14.71 | 17.736 |  |  | 4 | 28.593 | 25.515 | 28.671 |
| 8 | 36.759 | 32.54 | 33.978 |  |  | 8 | 41.426 | 44.26 | 47.592 |
